# Fall-Related Extremity Injuries During a Severe Snowfall and Icing Episode in Diyarbakır, Türkiye: Injury Patterns, Treatment Characteristics, and Need for Surgery in the Emergency Department

**DOI:** 10.3390/medicina62061152

**Published:** 2026-06-13

**Authors:** Mustafa Altintaş, Remzi Çetinkaya, Mehmet Özel, Habip Balsak

**Affiliations:** 1 Department of Orthopaedics and Traumatology, Diyarbakır Gazi Yaşargil Training and Research Hospital, University of Health Sciences, Diyarbakir 21070, Türkiye; drgoldstone4721@gmail.com; 2 Department of Emergency Medicine, Diyarbakır Gazi Yaşargil Training and Research Hospital, University of Health Sciences, Diyarbakir 21070, Türkiye; cetinkayaremzi2121@gmail.com; 3 Faculty of Health Sciences, Batman University, Batman 72000, Türkiye; h.balsak@gmail.com

**Keywords:** fall-related injury, snowfall, icing, extremity trauma, operative treatment, emergency department

## Abstract

*Background and Objectives*: Severe snowfall and icing are associated with weather-related trauma presentations, especially in cities unaccustomed to prolonged winter conditions. However, the clinical characteristics of these injuries and their implications for surgical management remain incompletely understood. This study aimed to describe injury patterns, treatment approaches, and factors associated with the need for surgery among patients presenting with extremity trauma during an intense snowfall and icing episode in Diyarbakır. *Materials and Methods*: This single-center retrospective observational study included patients presenting to the emergency department with extremity trauma during a severe snowfall and icing period. Demographic characteristics, injury features, imaging modality, ambient temperature, anatomical localization, and treatment approaches were analyzed. Patients were categorized according to nonoperative versus operative management. Factors associated with the need for surgery were evaluated using univariable and multivariable logistic regression analyses. Receiver operating characteristic analysis was used to assess the discriminative ability of age and ambient temperature for predicting the need for surgery. *Results*: A total of 943 patients were included. The largest age group was 18–44 years (38.6%), and 55.9% were male. Fractures were identified in 50.7% of cases, whereas 46.7% had no fracture and 2.7% had joint dislocation. Upper-extremity injuries predominated (65.2%), with distal segment involvement observed in 55.0% of cases. Most presentations occurred on days with mean ambient temperatures ≤ 0 °C (81.5%). Overall, 82.1% of patients were managed nonoperatively, while 17.9% required surgical treatment. In multivariable analysis, increasing age and the use of computed tomography were independently associated with the need for surgery, whereas ambient temperature was not. *Conclusions*: Fall-related extremity injuries during severe snowfall and icing were predominantly upper-extremity and distal injuries, and most were managed nonoperatively. The need for surgery was more strongly associated with patient age and injury complexity than with ambient temperature alone. These findings describe a distinct trauma profile during short-term winter events in mild-climate cities.

## 1. Introduction

Extreme weather events are increasingly recognized as an important public health concern due to their impact on healthcare systems and injury patterns. Climate change has been associated with alterations in the frequency and intensity of extreme meteorological conditions, including both heat- and cold-related events [1]. While the health effects of heat waves, floods, and storms have been widely studied, less attention has been given to the consequences of sudden snowfall and icing episodes, particularly in regions not typically exposed to prolonged winter conditions [2].

Although discussions of climate-related health effects often focus on heat waves, floods, and storms, cold-weather events and sudden snowfall episodes may also result in substantial secondary morbidity, particularly in cities that are not routinely exposed to prolonged snow and ice conditions [3]. In such settings, transportation disruption, inadequate pedestrian safety, untreated pavements, and widespread icing may contribute to slip-related falls and extremity trauma. This issue is especially relevant in relatively mild-climate urban centers, where both infrastructure and population behavior may be less adapted to severe winter conditions [4]. As a result, emergency departments may experience a sudden increase in trauma presentations, most commonly involving fall-related extremity injuries.

The province of Diyarbakır provides a relevant regional context for examining this issue, as an unusually severe winter episode affected a city not typically accustomed to prolonged snow exposure. During late December 2025 and early January 2026, Diyarbakır experienced an unusual snowfall episode that was locally reported to reach approximately 30–40 cm in the city center and was followed by marked icing, disrupting daily life, pedestrian mobility, and transportation [5]. Contemporary statements from Diyarbakır Metropolitan Municipality also documented city-wide snow removal, salting, and road-opening operations across central districts and major transportation routes, suggesting that the event represented a broad municipal operational challenge rather than a routine winter episode [6].

The relationship between snow-, ice-, and weather-related conditions and fracture epidemiology has been demonstrated in several populations. Studies from Canada and Ontario have shown that winter weather and icy conditions are associated with increased emergency department visits for falls and weather-dependent surges in orthopedic trauma volume [7,8]. Likewise, large registry-based studies from Sweden and the United States have reported that slipping on ice or snow is strongly associated with fractures, particularly around the wrist, forearm, and ankle, and that slippery weather may significantly increase distal radius fracture incidence [9,10]. However, these studies have largely been conducted in regions with well-established winter climates.

In contrast, relatively little is known about the clinical characteristics and management of such injuries in mild-climate cities experiencing sudden and severe winter events. From a biomechanical perspective, these injury patterns are also plausible. Slip-related falls commonly produce reflex protective extension of the upper extremity, resulting in distal radius, forearm, and hand injuries, whereas lower-extremity trauma may become more prominent in older adults and in higher-energy or unstable fall mechanisms [10]. Moreover, while the relationship between weather conditions and injury incidence is well documented, fewer studies have explored which patient- and injury-related factors are associated with the need for surgical management during these short-term but intense events. This issue is clinically relevant because weather-related trauma presentations may create substantial demands for trauma assessment, imaging, immobilization, and orthopedic consultation, even when operative treatment is required in only a subset of patients [11].

Therefore, the aim of this study was to describe the clinical characteristics of fall-related extremity injuries, to analyze treatment approaches, and to identify factors associated with the need for surgery among patients presenting to the emergency department during a severe snowfall and icing episode in Diyarbakır.

## 2. Materials and Methods

### 2.1. Study Design and Ethical Approval

This study was designed as a single-center, retrospective observational study. Data were obtained from the electronic medical records of patients presenting to the emergency department of a tertiary care hospital between 29 December 2025 and 18 January 2026, corresponding to a period of severe snowfall and widespread icing that significantly affected daily mobility in the region.

Ethical approval was obtained from the Institutional Clinical Research Ethics Committee of the University of Health Sciences Diyarbakır Gazi Yaşargil Training and Research Hospital (decision no: 42; date: 27 January 2026). The study was conducted in accordance with the principles of the Declaration of Helsinki. Due to the retrospective design, the requirement for informed consent was waived. All data were collected from anonymized patient records to ensure confidentiality.

### 2.2. Study Population

The study included all patients presenting to the emergency department with trauma during the defined snowfall and icing period who underwent radiological evaluation for suspected orthopedic injury. All eligible patients were included regardless of age. The inclusion criteria were presentation to the emergency department during the predefined study period because of trauma, radiological assessment with plain radiography alone or plain radiography combined with computed tomography for suspected orthopedic injury, and complete availability of treatment-related data in the medical records. In cases of repeated admissions, only the first presentation was included in the analysis. Patients with incomplete clinical, radiological, or treatment data were excluded.

### 2.3. Data Collection and Variable Definitions

Patient data were retrospectively retrieved from the hospital information management system and electronic medical records. The variables analyzed in the study included age, sex, type of injury, side of injury, affected extremity, imaging modality, ambient temperature on the day of injury, injury segment, fracture segment, affected anatomical region or major skeletal structure, treatment approach, and, in patients who underwent operative treatment, the operative technique used.

For descriptive and comparative analyses, age was categorized a priori into four clinically relevant groups: children/adolescents (0–17 years), young adults (18–44 years), middle-aged adults (45–64 years), and older adults (≥65 years). This grouping was selected to separate pediatric/adolescent patients from adults and to distinguish younger, middle-aged, and older adult trauma populations. Comparable adult age strata have been used in previous fall-related injury research, particularly 18–44, 45–64, and ≥65 years, and the ≥65-year threshold is widely used to define older trauma populations in trauma epidemiology studies [12]. For receiver operating characteristic (ROC) analysis, age was treated as a continuous variable. Injury type was classified according to the final radiological and orthopedic assessment as fracture, joint dislocation, or no radiologically confirmed fracture/dislocation. The latter group included patients with extremity trauma in whom imaging did not demonstrate fracture or dislocation and who were managed as soft-tissue injury, contusion, or sprain according to the emergency department record. This pragmatic classification was used because the main clinical aim was to distinguish fracture-/dislocation-oriented orthopedic injuries from radiologically negative trauma presentations. Injuries were grouped as upper-extremity or lower-extremity injuries. Imaging modality was categorized as plain radiography alone or plain radiography combined with computed tomography.

Ambient temperature data were obtained from the Turkish State Meteorological Service. Daily mean temperature values recorded by the meteorological station representing the city center of Diyarbakır were used for the meteorological assessment [13]. For group comparisons and logistic regression analyses, daily mean ambient temperature was categorized as ≤0 °C or ≥1 °C. For ROC analysis, ambient temperature was analyzed as a continuous variable.

Injury segment was classified for the overall cohort as proximal, diaphyseal, or distal. Fracture segment was evaluated only among patients with radiologically confirmed fractures and was similarly categorized as proximal, diaphyseal, or distal. The affected anatomical region or major skeletal structure was categorized as clavicle, arm/humerus, forearm/radius–ulna, hand region, pelvis/hip region, thigh/femur, leg/tibia–fibula, foot region, shoulder dislocation, or other. This classification was used to provide a clinically interpretable regional overview of injury distribution rather than a bone-by-bone anatomical inventory. Pelvic and hip-region injuries were retained in the analysis because they were evaluated within the orthopedic trauma workflow and were clinically relevant to operative treatment demand during the event period.

### 2.4. Outcome Measures

The primary outcome of the study was the need for surgical treatment. Treatment approaches were categorized as nonoperative or operative. Operative management included internal fixation with plates and screws, intramedullary nailing, arthroplasty, and other surgical procedures.

### 2.5. Statistical Analysis

Statistical analyses were performed using IBM SPSS Statistics for Windows, version 27.0 (IBM Corp., Armonk, NY, USA). Categorical variables were presented as number and percentage, n (%). Group comparisons were performed using Pearson’s chi-square test. When expected cell counts were insufficient, the Fisher–Freeman–Halton exact test or Monte Carlo correction was applied, as appropriate.

Patients were first categorized according to treatment approach as nonoperative or operative. To explore factors associated with the need for surgical management, logistic regression analyses were performed. Initially, a univariable logistic regression model was constructed using ambient temperature as the independent variable, given its potential role as an environmental risk indicator. Subsequently, a multivariable logistic regression model was constructed including age group, extremity involvement, and imaging modality, selected based on clinical relevance and univariable analysis. Collinearity diagnostics were performed for the independent variables included in the multivariable model, and no problematic multicollinearity was detected, as all tolerance values were >0.20 and all variance inflation factor values were <5.0. Given that imaging modality may reflect underlying injury complexity rather than act as an independent determinant of surgical decision-making, it was interpreted as a surrogate marker of injury severity within the model. Accordingly, the regression analysis was designed to identify variables associated with the likelihood of surgical management, rather than to establish causal relationships. Operative treatment status was defined as the dependent variable.

Among patients who required surgical intervention, clinical characteristics were further stratified according to the type of operative procedure performed. Receiver operating characteristic (ROC) curve analysis was also conducted to assess the ability of age and ambient temperature to predict the need for surgery. The area under the curve (AUC), optimal cut-off values, sensitivity, and specificity were calculated. A two-sided *p*-value < 0.05 was considered statistically significant.

## 3. Results

A total of 943 patients were included in the study. The cohort was predominantly composed of adults aged 18–44 years and male patients. Fracture was the most common final radiological diagnosis, upper-extremity and distal-region injuries predominated, and forearm/radius–ulna involvement was the leading anatomical localization. Most patients presented on days with mean ambient temperatures ≤0 °C and were treated nonoperatively. Detailed descriptive characteristics are presented in Table 1.

Comparisons according to treatment approach are presented in Table 2. Age distribution differed significantly between the nonoperative and operative treatment groups (*p* < 0.001). The proportion of operative treatment increased progressively with age, from 8.4% in the 0–17-year group to 12.1% in the 18–44-year group, 24.2% in the 45–64-year group, and 39.7% in the ≥65-year group. By contrast, sex distribution did not differ significantly between treatment groups (*p* = 0.969). Extremity localization was significantly associated with treatment approach (*p* < 0.001), with operative treatment being more frequent in lower-extremity injuries than in upper-extremity injuries (32.6% vs. 10.1%). Imaging modality also differed significantly between groups (*p* < 0.001). The proportion of operative treatment was 10.8% among patients evaluated with plain radiography alone, compared with 35.3% among those who underwent computed tomography in addition to plain radiography. The distribution of affected anatomical region or major skeletal structure was also significantly associated with treatment approach (*p* < 0.001). The highest operative treatment rate was observed in thigh/femur injuries (77.6%), followed by leg/tibia–fibula (33.0%), arm/humerus (21.8%), and pelvis/hip region injuries (20.0%). By contrast, the rates of operative treatment were lower in forearm/radius–ulna (11.3%), hand region (0.9%), and foot region injuries (1.5%). No statistically significant differences were observed between treatment groups with respect to injury segment (*p* = 0.273), side of injury (*p* = 0.241), or ambient temperature category (*p* = 0.476). In the fracture-only subgroup, fracture segment was not significantly associated with treatment approach (*p* = 0.306); operative treatment rates were 40.9% for proximal fractures, 30.8% for diaphyseal fractures, and 34.4% for distal fractures.

The results of logistic regression analysis for factors associated with operative treatment are shown in Table 3. In univariable analysis, ambient temperature was not significantly associated with the need for surgery. Compared with days with mean temperatures ≤ 0 °C, presentations on days with temperatures ≥ 1 °C were associated with a lower, but non-significant, likelihood of operative treatment (OR 0.854, 95% CI 0.548–1.331; *p* = 0.486). In the multivariable logistic regression model, ambient temperature remained non-significant, although a borderline association was observed (OR 0.575, 95% CI 0.329–1.005; *p* = 0.052). Age group was independently associated with operative treatment (*p* < 0.001). Using the 0–17-year group as the reference category, the 18–44-year group did not show a statistically significant increase in likelihood of operative treatment (OR 1.675, 95% CI 0.855–3.283; *p* = 0.133), whereas the 45–64-year group (OR 3.529, 95% CI 1.811–6.879; *p* < 0.001) and the ≥65-year group (OR 3.930, 95% CI 1.904–8.110; *p* < 0.001) were significantly more likely to undergo surgery. Extremity localization did not remain independently associated with operative treatment in the multivariable model (*p* = 0.132), despite a numerically higher likelihood for lower-extremity injuries (OR 11.240, 95% CI 0.481–262.828). By contrast, imaging modality was independently associated with operative treatment (*p* < 0.001). Patients evaluated with computed tomography in addition to plain radiography had an approximately 5.4-fold higher likelihood of undergoing surgery than those assessed with plain radiography alone (OR 5.396, 95% CI 3.494–8.332). The overall explanatory power of the multivariable model was moderate (Nagelkerke R^2^ = 0.439).

Clinical characteristics according to operative method among patients who underwent operative treatment are presented in Table 4. Among the 169 patients who underwent surgery, plate–screw fixation was the most frequently used technique (70.4%), followed by intramedullary nailing (18.3%), arthroplasty (7.1%), and other operative procedures (4.1%). The distribution of operative methods did not differ significantly according to ambient temperature (*p* = 0.708). However, significant differences were observed according to age group (*p* < 0.001). Plate–screw fixation predominated in the 18–44-year and 45–64-year groups, accounting for 86.4% and 81.4% of procedures, respectively. In contrast, intramedullary nailing and arthroplasty were relatively more common in patients aged ≥65 years, with rates of 31.3% and 18.8%, respectively. In the 0–17-year group, other operative procedures accounted for 22.2% of operations. Operative method also differed significantly by imaging modality (*p* < 0.001). Among patients assessed with plain radiography alone, plate–screw fixation was performed in 43.1%, intramedullary nailing in 38.9%, and arthroplasty in 11.1%. In contrast, among patients evaluated with computed tomography in addition to plain radiography, plate–screw fixation was overwhelmingly predominant (90.7%). The affected anatomical region or major skeletal structure was also significantly associated with operative method (*p* < 0.001). Plate–screw fixation was the dominant procedure in forearm/radius–ulna injuries (97.6%), arm/humerus injuries (84.2%), pelvis/hip-region injuries (100.0%), and leg/tibia–fibula injuries (78.5%). In thigh/femur injuries, however, the distribution of operative methods differed substantially: intramedullary nailing was used in 47.4%, arthroplasty in 31.6%, and plate–screw fixation in 18.4% of cases.

The discriminative performance of age and ambient temperature for predicting operative treatment was evaluated using ROC analysis, and the results are presented in Table 5. Age demonstrated a statistically significant but limited discriminatory performance for operative treatment (AUC 0.695, 95% CI 0.651–0.739; *p* < 0.001). The optimal cutoff value for age was 33.5 years, yielding a sensitivity of 77.5% and a specificity of 49.2%. By contrast, ambient temperature did not show significant discriminatory ability for predicting the need for surgery (AUC 0.525, 95% CI 0.480–0.569; *p* = 0.312). Therefore, no clinically meaningful cutoff value was identified for ambient temperature.

## 4. Discussion

This study evaluated the clinical characteristics, treatment patterns, and factors associated with the need for surgery among patients presenting to the emergency department with orthopedic trauma during a severe snowfall and icing episode in Diyarbakır. Most presentations occurred on days with ambient temperatures ≤0 °C, and injuries predominantly affected the upper extremity and distal segments. Radius–ulna injuries were the most frequently observed, and most patients were managed nonoperatively. The need for surgical intervention was more strongly associated with older age, lower-extremity involvement (particularly femoral injuries), and the use of computed tomography, whereas ambient temperature alone was not independently associated with surgical requirement.

The finding that most orthopedic injuries occurred during periods with ambient temperatures at or below 0 °C is consistent with previous reports showing that cold weather, snow, and icy conditions are associated with increased trauma-related emergency department visits, particularly those related to slips and falls [7,8]. In our cohort, the predominance of upper-extremity injuries and the high frequency of radius–ulna involvement are also in line with the typical injury pattern described in the literature for fall events on snow or ice, in which protective extension of the upper extremity often results in distal forearm and wrist trauma [9,14]. Likewise, the predominance of distal segment involvement in both the overall cohort and the fracture subgroup is biomechanically plausible and further supports the view that these injuries were largely driven by slip-related low-energy mechanisms [9].

Most injuries observed during the snowfall and icing period were managed nonoperatively. The need for surgical treatment was more strongly associated with patient age, lower-extremity involvement, and the use of computed tomography, rather than with ambient temperature alone. These findings indicate that the clinical impact of weath weather-related trauma extends beyond surgical demand and encompasses a broader range of emergency department services, including imaging, immobilization, analgesia, discharge planning, and orthopedic consultation. However, several limitations should be acknowledged. In the absence of a pre-event or a seasonal or historical control group, this study cannot determine whether the snowfall and icing episode resulted in an overall increase in trauma volume compared with baseline conditions.

The age and sex distribution in our cohort differed somewhat from the classic epidemiological profile of distal forearm fractures, which are often reported more frequently in older women, especially after the age of 50 years and during winter months [9,14]. In our cohort, the most affected age group was 18–44 years, and men were slightly more represented than women. This distribution suggests that injury patterns during sudden snowfall and icing events may differ from the classical osteoporotic fracture profile. Instead, increased mobility, occupational exposure, commuting demands, and greater outdoor activity may have contributed to the higher representation of younger and middle-aged adults. However, because these factors were not directly assessed, this interpretation should be regarded as hypothesis-generating rather than definitive.

Older age was associated with a higher likelihood of operative treatment; however, this finding should be interpreted in operational rather than strictly biological terms. During this short-term snowfall and icing episode, younger and middle-aged adults accounted for many emergency department presentations, probably reflecting greater outdoor exposure, commuting, and occupational mobility. In contrast, older patients were disproportionately represented among operative cases, mainly because fall-related trauma in this group more frequently involved femoral, hip-region, or other load-bearing lower-extremity injuries requiring fixation or arthroplasty [15,16,17,18]. Similarly, the association between computed tomography use and operative treatment should not be interpreted as causal. CT was more likely requested in patients with suspected complex fractures, intra-articular involvement, comminution, or injuries requiring preoperative planning; therefore, it should be regarded as a surrogate marker of injury complexity [19]. The wide confidence interval for lower-extremity involvement in the multivariable model further supports the need for cautious interpretation, suggesting limited precision and possible model instability rather than a definitive independent effect of broad extremity location.

Although lower-extremity injuries were associated with a higher crude rate of surgery than upper-extremity injuries, this relationship did not remain statistically significant in the multivariable model. This suggests that operative decision-making may be driven less by broad extremity location and more by specific fracture type, load-bearing function, and the need to restore alignment and mobility. In our cohort, thigh/femur injuries had the highest operative treatment rate, and leg/tibia–fibula injuries also showed a relatively high proportion of operative treatment. These findings are clinically expected, as femoral fractures often require intramedullary fixation or arthroplasty depending on fracture type, whereas tibia–fibula injuries—especially those involving distal tibia, malleolar, or ankle-adjacent structures—frequently require fixation to restore stability and function [15,16,17]. By contrast, upper-extremity injuries were predominantly treated nonoperatively, which is consistent with the generally nonoperative management of many low-energy distal radius, clavicle, and hand fractures [20]. Humerus and radius–ulna injuries represented exceptions, with plate–screw fixation predominating in operative cases, in line with established surgical practice for selected humeral fractures and adult both-bone forearm fractures [21,22].

Ambient temperature, despite its relevance to the environmental context of trauma presentations, was not independently associated with the need for surgery. This finding should not be interpreted as evidence that weather severity is clinically irrelevant; rather, daily mean ambient temperature alone may be insufficient to capture actual slip risk or injury severity. ROC analysis similarly showed that ambient temperature had no meaningful discriminatory performance, whereas age had statistically significant but limited discriminatory ability. The age threshold identified by ROC analysis should not be regarded as a clinically actionable cutoff, but as a statistical reflection of the increasing proportion of fracture types treated operatively with age.

Overall, our findings describe a trauma profile observed during a severe snowfall and icing period in a city not routinely exposed to prolonged winter conditions. This profile was characterized mainly by low-energy extremity injuries, most of which were managed nonoperatively, together with a smaller subgroup of more complex injuries requiring surgery.

### Limitations

This study has several limitations. First, its retrospective and single-center design limits causal inference and may reduce the generalizability of the findings to other centers or regions with different climatic conditions. Second, the study included only patients presenting during the predefined snowfall and icing period and did not include a pre-event, seasonal, or historical control group. Therefore, this study cannot quantify the magnitude of any increase in emergency department trauma volume attributable to the snowfall and icing episode. The findings should be interpreted as a descriptive profile of trauma presentations observed during the event period rather than as direct evidence that snowfall independently increased trauma incidence or operative workload.

Third, because the data were obtained from retrospective records, potentially relevant variables such as the exact mechanism of injury, individual environmental exposure, comorbidities, osteoporosis status, and baseline functional status could not be analyzed in detail. Fracture-level radiological details such as displacement, intra-articular extension, comminution, fracture stability, and exact fracture classification were not consistently available in the retrospective records. The absence of these variables may have limited the ability of the multivariable model to capture the true determinants of surgical decision-making, because operative treatment is often influenced by fracture morphology rather than by broad anatomical region alone. Similarly, the no-fracture group was not subdivided into sprain, contusion, ligament injury, or other soft-tissue diagnoses because these subcategories were inconsistently documented in retrospective emergency department records. In addition, daily mean ambient temperature was the only meteorological variable analyzed and may not fully reflect true environmental slip risk. Factors such as snowfall accumulation, precipitation intensity, humidity, freeze–thaw cycles, surface icing, snow clearance, salting practices, pedestrian density, and time-of-day temperature variation were not available. Therefore, the lack of association between ambient temperature and operative treatment should not be interpreted as evidence that weather severity is clinically irrelevant. The association between computed tomography use and operative treatment should also be interpreted as a marker of suspected injury complexity rather than a causal relationship. Furthermore, the wide confidence interval observed for lower-extremity involvement in the multivariable model indicates limited precision and possible model instability; therefore, this estimate should be interpreted cautiously. Finally, long-term functional outcomes, complications, and rehabilitation requirements were not evaluated.

## 5. Conclusions

During a severe snowfall and icing episode in Diyarbakır, extremity trauma presentations were mainly characterized by upper-extremity, distal-region, and forearm/radius–ulna injuries, and most patients were managed nonoperatively. Although most presentations occurred on days with mean ambient temperatures ≤ 0 °C, ambient temperature alone did not independently predict the need for surgery. Operative treatment was concentrated in older patients and in patients with anatomically complex or lower-extremity injuries requiring advanced imaging. These findings suggest that, in cities not routinely exposed to prolonged snow and ice, short-term winter events may be associated with a characteristic pattern of emergency department orthopedic trauma presentations, in which most injuries are managed nonoperatively while a smaller but clinically important subgroup requires surgery.

## Figures and Tables

**Table 1:** Descriptive characteristics of extremity trauma patients presenting during the heavy snowfall period.

Variables	n	%	Variables	n	%
**Age**			**Injury segment**		
0–17 years	214	22.7	Proximal	257	27.3
18–44 years	364	38.6	Diaphyseal	167	17.7
45–64 years	244	25.9	Distal	519	55.0
≥65 years	121	12.8	**Fracture segment**		
**Sex**			Proximal	115	24.1
Female	416	44.1	Diaphyseal	78	16.3
Male	527	55.9	Distal	285	59.6
**Type of injury**			**Affected anatomical region or major skeletal structure**		
Fracture	478	50.7	Clavicle	15	1.6
No fracture	440	46.7	Arm/humerus	87	9.2
Joint dislocation	25	2.7	Forearm/radius–ulna	372	39.4
**Side**			Hand region	107	11.3
Right	457	48.5	Pelvis/hip region	15	1.6
Left	486	51.5	Thigh/femur	49	5.2
**Extremity**			Leg/tibia–fibula	197	20.9
Upper extremity	615	65.2	Foot region	66	7.0
Lower extremity	328	34.8	Shoulder dislocation	20	2.1
**Imaging modality**			Other	15	1.6
Plain radiography	668	70.8	**Treatment status**		
CT + plain radiography	275	29.2	**Nonoperative**	774	82.1
**Ambient temperature**			Operative	169	17.9
≤0 °C	769	81.5	**Operative method**		
≥1 °C	174	18.5	Plate–screw fixation	119	70.4
			Intramedullary nail fixation	31	18.3
			Arthroplasty	12	7.1
			Other	7	4.1

Data are presented as n (%). Unless otherwise stated, percentages were calculated based on the total number of patients (N = 943). Fracture segment was calculated only among patients with fractures (n = 478). Operative method was calculated only among patients who underwent operative treatment (n = 169). CT: computed tomography.

**Table 2:** Comparison of demographic and clinical characteristics according to treatment approach.

Variables	Nonoperative, n (%)	Operative, n (%)	*p*
**Age**			**<0.001**
0–17 years	196 (91.6)	18 (8.4)	
18–44 years	320 (87.9)	44 (12.1)	
45–64 years	185 (75.8)	59 (24.2)	
≥65 years	73 (60.3)	48 (39.7)	
**Sex**			0.969
Female	341 (82.0)	75 (18.0)	
Male	433 (82.2)	94 (17.8)	
**Extremity**			**<0.001**
Upper extremity	553 (89.9)	62 (10.1)	
Lower extremity	221 (67.4)	107 (32.6)	
**Imaging modality**			**<0.001**
Plain radiography	596 (89.2)	72 (10.8)	
CT + plain radiography	178 (64.7)	97 (35.3)	
**Injury segment**			0.273
Proximal	211 (82.1)	46 (17.9)	
Diaphyseal	144 (86.2)	23 (13.8)	
Distal	419 (80.7)	100 (19.3)	
**Side**			0.241
Right	382 (83.6)	75 (16.4)	
Left	392 (80.7)	94 (19.3)	
**Ambient temperature**			0.476
≤0 °C	628 (81.7)	141 (18.3)	
≥1 °C	146 (83.9)	28 (16.1)	
**Affected anatomical region or major skeletal structure**			**<0.001**
Clavicle	15 (100.0)	0 (0.0)	
Arm/humerus	68 (78.2)	19 (21.8)	
Forearm/radius–ulna	330 (88.7)	42 (11.3)	
Hand region	106 (99.1)	1 (0.9)	
Pelvis/hip region	12 (80.0)	3 (20.0)	
Thigh/femur	11 (22.4)	38 (77.6)	
Leg/tibia–fibula	132 (67.0)	65 (33.0)	
Foot region	65 (98.5)	1 (1.5)	
Shoulder dislocation	20 (100.0)	0 (0.0)	
Other	15 (100.0)	0 (0.0)	
**Fracture segment among patients with fractures only**			0.306
Proximal	68 (59.1)	47 (40.9)	
Diaphyseal	54 (69.2)	24 (30.8)	
Distal	187 (65.6)	98 (34.4)	

Data are presented as n (%). Percentages are row percentages. Nonoperative and operative treatment groups included 774 and 169 patients, respectively, in the overall population. Fracture segment analysis was performed only among patients with fractures, including 309 nonoperatively treated and 169 operatively treated patients. CT: computed tomography.

**Table 3:** Univariable and multivariable logistic regression analysis of factors associated with the need for surgery.

Model	Variables	B	OR (95% CI)	*p*
**Univariable model**	**Ambient temperature**			0.486
	≤0 °C		Reference	
	≥1 °C	−0.158	0.854 (0.548–1.331)	
**Multivariable model**	**Ambient temperature**			0.052
	≤0 °C		Reference	
	≥1 °C	−0.553	0.575 (0.329–1.005)	
	**Age**			**<0.001**
	0–17 years		Reference	
	18–44 years	0.516	1.675 (0.855–3.283)	0.133
	45–64 years	1.261	3.529 (1.811–6.879)	**<0.001**
	≥65 years	1.369	3.930 (1.904–8.110)	**<0.001**
	**Extremity**			0.132
	Upper extremity		Reference	
	Lower extremity	2.419	11.240 (0.481–262.828)	
	**Imaging modality**			**<0.001**
	Plain radiography		Reference	
	CT + plain radiography	1.686	5.396 (3.494–8.332)	

Nagelkerke R^2^ was 0.001 for the univariable model and 0.439 for the multivariable model. B: regression coefficient; OR: odds ratio; CI: confidence interval; CT: computed tomography.

**Table 4:** Comparison of clinical characteristics according to operative method among patients who underwent operative treatment.

Variables	Plate and Screw Fixation (n = 119), n (%)	Intramedullary Nailing (n = 31), n (%)	Arthroplasty (n = 12), n (%)	Other Operative Methods (n = 7), n (%)	*p*
**Ambient temperature**					0.708
≤0 °C	98 (69.5)	27 (19.1)	11 (7.8)	5 (3.5)	
≥1 °C	21 (75.0)	4 (14.3)	1 (3.6)	2 (7.1)	
**Age group**					<0.001
0–17 years	10 (55.6)	4 (22.2)	0 (0.0)	4 (22.2)	
18–44 years	38 (86.4)	5 (11.4)	0 (0.0)	1 (2.3)	
45–64 years	48 (81.4)	7 (11.9)	3 (5.1)	1 (1.7)	
≥65 years	23 (47.9)	15 (31.3)	9 (18.8)	1 (2.1)	
**Imaging modality**					<0.001
Plain radiography	31 (43.1)	28 (38.9)	8 (11.1)	5 (6.9)	
CT + plain radiography	88 (90.7)	3 (3.1)	4 (4.1)	2 (2.1)	
**Affected anatomical region or major skeletal structure**					<0.001
Arm/humerus	16 (84.2)	0 (0.0)	0 (0.0)	3 (15.8)	
Forearm/radius–ulna	41 (97.6)	0 (0.0)	0 (0.0)	1 (2.4)	
Hand region	1 (100.0)	0 (0.0)	0 (0.0)	0 (0.0)	
Pelvis/hip region	3 (100.0)	0 (0.0)	0 (0.0)	0 (0.0)	
Thigh/femur	7 (18.4)	18 (47.4)	12 (31.6)	1 (2.6)	
Leg/tibia–fibula	51 (78.5)	13 (20.0)	0 (0.0)	1 (1.5)	
Foot region	0 (0.0)	0 (0.0)	0 (0.0)	1 (100.0)	

Data are presented as n (%). Percentages are row percentages. The analysis was performed only among patients who underwent operative treatment (n = 169). CT: computed tomography. *p* values indicate comparisons across operative method groups. Note. Collinearity diagnostics indicated no problematic multicollinearity among the independent variables included in the multivariable logistic regression model, as all tolerance values were >0.20 and all variance inflation factor (VIF) values were <5.0.

**Table 5:** ROC analysis of age and ambient temperature for predicting the need for surgery.

Outcome Variable	Predictor Variable	AUC (95% CI)	Cut-Off Value	Sensitivity	Specificity	*p*
Need for surgery	Age	0.695 (0.651–0.739)	33.50 years	0.775	0.492	<0.001
Need for surgery	Ambient temperature	0.525 (0.480–0.569)	—	—	—	0.312

AUC: area under the curve; CI: confidence interval; ROC: receiver operating characteristic.

## Data Availability

The data supporting the findings of this study are available from the corresponding author upon reasonable request.
